# Tracing the genetic fingerprints of tumour evolution: The pursuit of identifying mutations with differential weights within the overall tumour mutation burden and their role in therapeutic responses with immune checkpoint blockade

**DOI:** 10.1002/ctm2.1287

**Published:** 2023-05-30

**Authors:** Noushin Niknafs, Michael Conroy, Valsamo Anagnostou

**Affiliations:** ^1^ The Sidney Kimmel Comprehensive Cancer Center Johns Hopkins University School of Medicine Baltimore Maryland; ^2^ The Bloomberg‐Kimmel Institute for Cancer Immunotherapy Johns Hopkins University School of Medicine Baltimore Maryland

1

Immune checkpoint blockade (ICB) has transformed the therapeutic paradigm of several cancers and is now FDA‐approved as monotherapy, combined anti‐PD‐(L)1/CTLA‐4 therapy, or in combination with chemotherapy for treatment of several tumour types including melanoma, lung cancer, urothelial cancer and head and neck cancer. Its scope has also expanded beyond the palliative treatment of advanced malignancies to neoadjuvant, adjuvant and consolidative use with curative intent.^1^ As the spectrum of immuno‐oncology (IO) treatment options further expands, it will be increasingly useful to implement biomarkers that allow us to stratify patients by risk of recurrence, and to guide treatment decisions accordingly.^2^ For example, patients with metastatic nonsmall cell lung cancer (NSCLC) may be more accurately matched to ICB monotherapy, PD‐(L)1/CTLA‐4 combination ICB or ICB/chemotherapy combinations depending on molecularly assessed risk of recurrence. Furthermore, despite significant and sustained responses, most patients receiving ICB treatment develop acquired resistance.^3^ Therefore, patient selection for these therapies represents a major clinical need that – with the exception of microsatellite instability – is not met by use of current predictive   biomarkers such as PD‐L1 expression or tumour mutation burden.^2^

**FIGURE 1:**
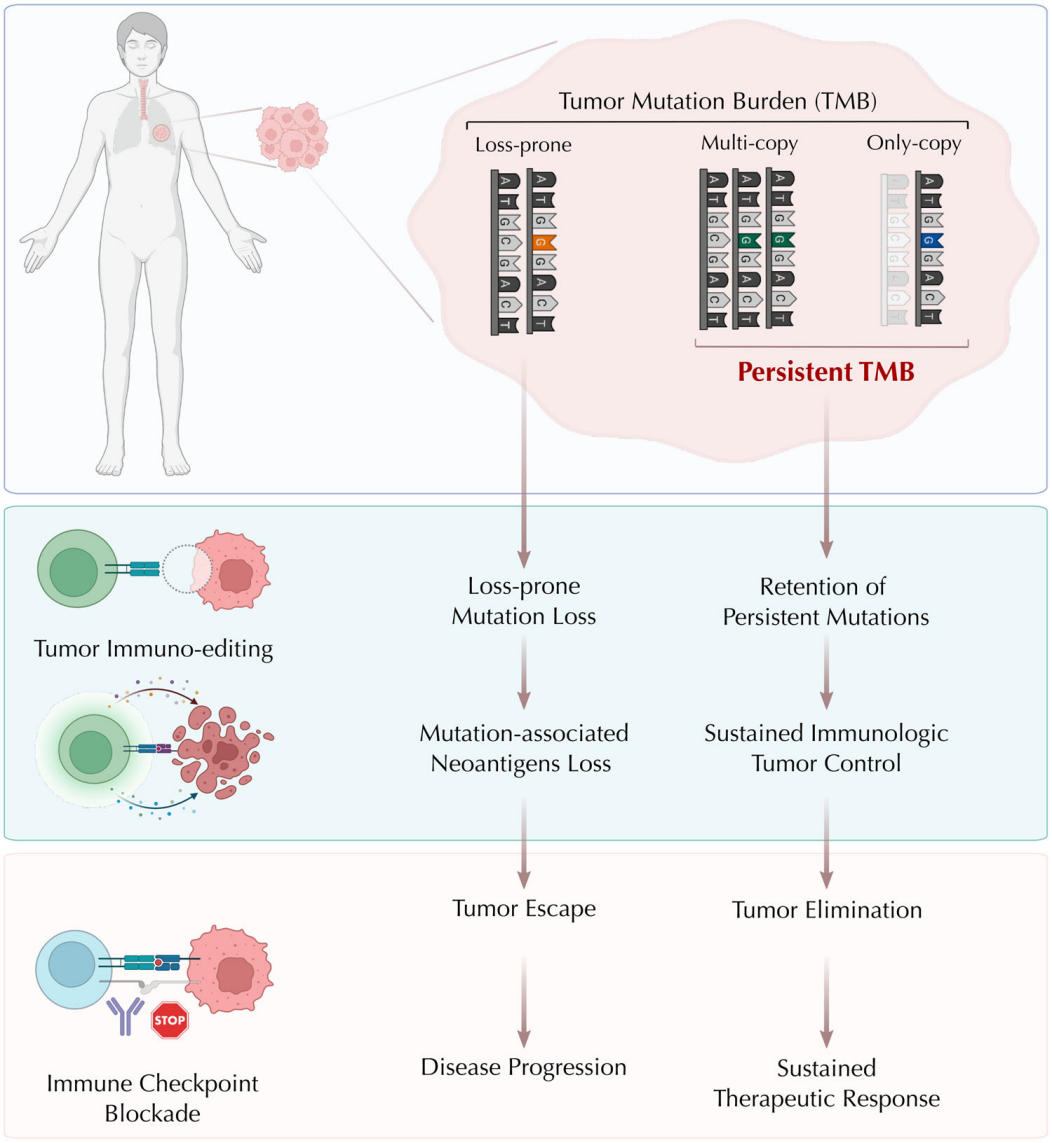
Persistent mutations represent an uneditable set within the overall TMB that may drive immunologic tumour control and sustained clinical therapeutic responses. Persistent mutations in cancer cells are resistant to tumour immune‐editing during the natural course of tumour evolution and contribute to sustained immunologic tumour control. During ICB treatment, tumours with high persistent mutation burden are more likely to regress due to their inability to evade immune recognition by mutation loss, resulting in sustained therapeutic response and favourable outcome. Conversely, loss‐prone mutations and their associated neoantigens are more likely to be eliminated in the process of tumour evolution. Therefore, tumours with predominantly loss‐prone mutations can undergo immune escape leading to disease progression in the context of therapy.

During the course of tumour evolution, cancer cells typically accumulate somatic sequence alterations, a fraction of which code for mutation‐associated neoantigens (MANA) that are presented on human leukocyte antigen (HLA) molecules to T cells, eliciting immune recognition in the context of mutant ‘nonself’ neopeptides. Immune checkpoint blockade unleashes a potent immune response against MANAs, that is amplified in the context of a high neoantigen burden. Conceptually, a higher neoantigen burden confers a higher degree of tumour foreignness to the immune system and clinically this is reflected in therapeutic benefit with ICB for patients with TMB‐high tumours. To this end, TMB has been utilised as a proxy for tumour foreignness however has been inconsistently associated with ICB response.^2^, ^4^ This is driven in part by challenges with the accurate estimation of TMB, especially in analysis of low tumour purity samples,^5^ absence of cancer lineage‐specific thresholds that define what TMB‐high tumours are and more importantly the lack of consideration of mutations with differential biological weights within the overall TMB.^6^

In studying the evolving lung cancer genomes under the selective pressure of immunotherapy, we identified loss of MANAs, via copy number loss or subclonal elimination, as one of the key genomic mechanisms underlying the emergence of acquired resistance.^7^ This observation brought focus to the contribution of somatic copy number alterations in the context of ICB response. Further comprehensive analyses of sequence and structural genomic landscapes of ICB‐treated mesotheliomas, that represent tumours lying in the lower end of the TMB spectrum, revealed that the density of mutations residing in haploid genomic regions is associated with therapeutic response.^8^ Collectively, these insights motivated an in‐depth inquiry around the notion of mutation loss in the context of ICB, and whether we can improve the utility of TMB by incorporating information capturing the propensity of tumours to undergo mutation loss and thus escape tumour control in the context of immunotherapy.

We hypothesised that mutations within the overall TMB fall within two categories: that of ‘loss‐prone’ and that of ‘persistent’ mutations (Figure 1).^9^ Persistent mutations signify sequence alterations that might be resistant to loss in cancer cells and these in turn fall within two subclasses. The first subclass of persistent mutations is defined as mutations in single‐copy (haploid) regions of the genome (only‐copy mutations). We reasoned that loss of these mutations could confer a fitness disadvantage to cancer cells via complete loss of essential gene function in linkage with persistent mutations. The second class of persistent mutations are those present in multiple copies per cancer cell (multicopy mutations), and loss of these mutations could only be achieved by multiple distinct genomic alterations that is evolutionarily less probable.

We set out to explore this hypothesis by computational analyses of ∼10 000 tumour samples from the cancer genome atlas (TCGA), and identified an extensive variation in the abundance of only‐copy and multicopy mutations between and within tumour types. On average, we found persistent mutations to be a small minority (10%) of the somatic mutations per tumour. Importantly, when we evaluated the differential reclassification of cancers across 33 tumour types, we found an average reclassification rate of 33% in TMB‐high/low versus persistent TMB‐high/low groups. These findings suggest that tumours are differentially classified based on their persistent tumour mutation burden (pTMB). To explore the clinical significance of these findings, we analysed 524 ICB‐treated tumour samples including head and neck cancer, mesothelioma, nonsmall cell lung cancer and melanoma. In all cohorts analysed, pTMB distinguished responding from nonresponding tumours better than TMB or the number of loss‐prone mutations.^9^ Notably, while representing a small fraction of the overall TMB, pTMB could differentiate response groups in tumour types where TMB failed to do so, posing the question whether pTMB could more optimally stratify patients more likely to attain therapeutic benefit with immunotherapy. In evaluating the dynamics of mutation loss under the selective pressure of ICB and consistent with the hypothesis of persistent mutations that are retained during tumour evolution, we confirmed a >60‐fold lower rate of loss of persistent mutations compared to loss‐prone mutations.

A key question that arose from these studies was whether persistent mutation burden can be computed from targeted next‐generation sequencing that is widely adopted in clinical practice. To assess the feasibility and clinical value of pTMB in routine cancer care, we performed in silico simulations, where we restricted the TMB and pTMB definitions to genomic regions queried by clinical grade targeted next‐generation sequencing and found that, similar to whole exome sequencing data, pTMB defined based on targeted NGS outperformed TMB in distinguishing responding from nonresponding tumours in the context of ICB. These findings, while requiring further validation, are encouraging with respect to the immediate clinical utility of persistent mutations. Historically, we have been focusing on the density of sequence alterations derived from tumour molecular profiling, ignoring the structural genomic landscape that can be reliably derived from targeted next‐generation sequencing. By integrating sequence and structural landscapes and considering sequence mutations in the context of the background copy number of the locus evaluated, we can maximally leverage next‐generation sequence data and tailor the tumour mutation burden to the subset of alterations most likely to drive immunologic tumour control.

In thinking about the clinical implementation of pTMB, there are several critical steps needed to bring consensus on the clinical significance of varying pTMB results in cancers of different lineages. Dichotomised biomarkers, such TMB ‘high’ or ‘low’, are simple and accessible, but it is increasingly clear that their universal predictive ability is limited. Conversely, overly granular metrics allow for nuance, but may not be practical for routine cancer care. While pTMB provides a composite measure for the number of hard‐to‐lose mutations in a tumour, only‐copy or multicopy subclasses may carry differential weight in predicting ICB response, potentially driven by the dominant copy number state of the tumour evaluated. For example, in the setting of a melanoma tumour, multicopy mutations may be most informative, whereas single‐copy alterations would be more predictive in ICB‐treated mesotheliomas. Understanding the relative importance of only‐copy and multicopy mutations across cancer lineages will help to further improve the clinical utility of pTMB. Furthermore, lineage‐specific pTMB thresholds, and stability of such thresholds require additional studies and validation.

As pTMB represents one piece in the jigsaw puzzle of tumour‐immune system interactions, it may fit within an integrative composite predictive tool together with tumour‐extrinsic features. Furthermore, placing these observations in the context of other measures of neoantigen quality,^10^ development of multifactorial models capturing distinct properties of neoantigens such as clonality, expression, MHC affinity, immunogenicity and persistence can enable accurate identification of the subset of neoantigens driving ICB response and thus improve risk stratification and inform patient selection strategies.

In the clinical setting, there are several scenarios in which pTMB may be particularly informative. One possibility is in situations where other biomarkers have conflicting results. For example, if a nonsmall cell lung cancer tumour is found to have high TMB but low PD‐L1, the pTMB result may support decision‐making: is there a strong chance of response to ICB monotherapy, or is combination with chemotherapy required? pTMB levels may also be informative for TMB‐high tumours which are less likely to have a treatment response at the standard ‘high’ cutoff (>10 muts/Mb), such as in breast cancer.

In conclusion, the expanding clinical uptake of ICB has not been matched by the development of accurate predictors of response. To this end, measurement of pTMB represents a promising and innovative approach to predicting response to ICB compared to the overall tumour mutation burden. Focusing on the subset of mutations within the overall TMB most likely to drive sustained immunologic tumour control can extend the promise of cancer immunotherapy and improve patient outcomes.

## CONFLICT OF INTEREST STATEMENT

V.A. receives research funding to Johns Hopkins University from Astra Zeneca, Personal Genome Diagnostics and Delfi Diagnostics, has received research funding from Johns Hopkins University from Bristol‐Myers Squibb in the past 5 years and is an advisory board member for Neogenomics. V.A. is an inventor on patent applications (63/276525, 17/779936, 16/312152, 16/341862, 17/047006 and 17/598690) submitted by Johns Hopkins University related to cancer genomic analyses, ctDNA therapeutic response monitoring and immunogenomic features of response to immunotherapy that have been licensed to one or more entities. Under the terms of these license agreements, the university and inventors are entitled to fees and royalty distributions.
